# Combined Femoral and Acetabular Osteotomy in Children of Walking Age for Treatment of DDH; A Five Years Follow-Up Report

**Published:** 2015-01

**Authors:** Mahdi Mazloumi, Farzad Omidi-Kashani, Mohamad Hosein Ebrahimzadeh, Hadi Makhmalbaf, Mohamad Mahdi Hoseinayee

**Affiliations:** Department of Orthopedic Surgery, Orthopedic Research Center, Ghaem Hospital, Mashhad University of Medical Sciences, Mashhad, Iran

**Keywords:** Developmental dysplasia of the hip, Osteotomy, Radiography

## Abstract

**Background:**

The prevalence of neglected developmental dysplasia of the hip (DDH) has been decreasing. Nowadays, the disease is rarely seen in walking age children. The purpose of this study is to assess the results of simultaneous osteotomy of femur and pelvic bones in such children.

**Method:**

We performed a retrospective study on 30 children aged 3.8±0.9 (range: 1.5-7) years old, with DDH who underwent surgical operation in our hospital from August 2001 to September 2006. Tönnis and Severin grading systems were used to classify the radiographic status of the hip in pre- and postoperative era, respectively. Improvement in function and limp was also evaluated by the modified McKay’s classification.

**Results:**

From the 30 cases, six patients excluded in the course of the study and among the remaining patients, 12 had bilateral involvement. The mean follow-up period was 7.6±0.8 (range: 5.1-11.3) years. During the last visit, radiographic status of the operated joints, according to Severin classification was as follows: Class I: 12 patients; Class II: 20 patients; Class III: 3 patients; Class IV: 1 patient; and Class VI: 1 patient.

**Conclusion:**

Although through the follow-up, two hips subluxated, necrosis happened in three and one joint was re-dislocated, simultaneous femoral and innominate osteotomy in the walking age children with DDH has relatively good clinical outcomes.

## Introduction

Developmental dysplasia of the hip (DDH) encompasses a spectrum of diseases that include hip instability, hip subluxation with underdeveloped acetabulum and incomplete coverage of the femoral head and hip dislocation.^1^ Pain occurs earlier in the child with hip subluxation than with dislocation.^2^ Untreated hip dysplasia can lead to early onset arthritis, joint destruction and ultimately the need for artificial joint replacement which adversely affect patient’s quality of life.^3^^,^^4^ In those cases who are refractory to closed reduction, early open reduction to reorient the acetabulum in order to maintain the physiologic growth of the hip joint must be surgically addressed.^5^ Thomas et al., in a long-term follow-up study at 40-45 years after open reduction and innominate osteotomy for late presenting DDH, reported an excellent prognosis in two third of the patients.^6^Favorable coverage of the femoral head was reported to be obtained gradually by shifting the center of the femoral head caudally and medially as well as rotating the distal fragment anterolaterally.^7^

Open reduction and innominate osteotomy are two appropriate surgical techniques that are necessary in the initial management of the children with late presentation.^8^^-^^10^ In children more than two years of age who walk on the dislocated joint, in the case of persistent excessive anteversion of the femoral neck and femoral head displacement of more than one-third of the iliac width, both femoral and innominate osteotomies should be considered.^11^^,^^12^ Femoral osteotomy combined with innominate osteotomy is usually performed to prevent excessive pressure on the head, causing osteonecrosis. This is also done in older children with severe anteversion. Reorientation of the acetabulum which causes an anterior coverage of the femoral head may itself increase risk of posterior subluxation or dislocation of the joint after the operation.^13^ These types of surgery in older children are associated with more numerous complications such as re-dislocation, avascular necrosis (AVN) of the femoral epiphysis, and ultimately femoral neck varus and limp.^14^^-^^16^

Nowadays, with the improvements achieved in the diagnosis and screening of DDH, the prevalence of late presentations has decreased significantly and the disease is rarely discovered in the walking age children. The purpose of this study is to report the results of the open reduction and concomitant femoral and innominate osteotomy in the treatment of DDH in the walking age children. 

## Patients and Methods

After obtaining local review board approval and assignment of the informed consent form by at least one of the parents, we performed a retrospective study on 24 children of walking age involved with DDH who underwent surgical operation in our hospital from August 2001 to September 2006. Half of them had bilateral DDH. Before and after surgery, radiographic classification according to Tönnis and Severin classification system were used, respectively.^17^^,^^18^ Tönnis classification is usually used for evaluating osteoarthritis by radiographic changes (Grade 0 to 3), and Severin classification is commonly used to assess the radiographic results of operations carried out for the treatment of DDH.

Radiographic control study was assessed before, after surgery, and at 6 months, one and two years, and final visits. The inclusion criterion was children between 1.5-7 years old who can walk. Exclusion criteria were previous surgery on the involved hip, children less than 1.5 years of age, and teratologic or neuromuscular hip dysplasia with difficult walking ability. Improvement in joint mobility and limp at final follow-up visit was assessed by the modified McKay classification.^19^ This classification is especially useful for assessing the clinical results.

*Surgical Technique*

**Figure 1:**
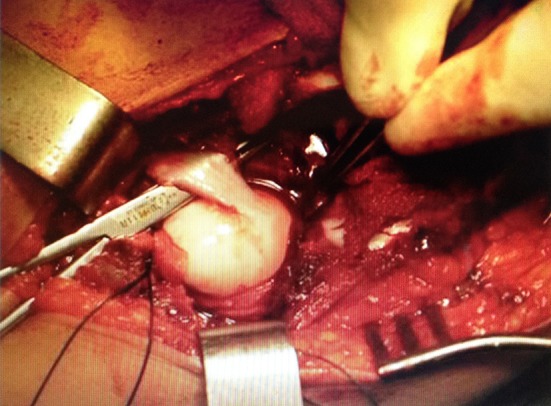
Intra-operative picture showing the dislocated hip and the round ligament just before resection.

At first, adductor tenotomy was performed to release muscle contractures in the adductor muscles group**. **Hip arthrotomy was performed using the iliofemoral approach and then the iliac crest apophysis was divided into two halves in order to gain access to the joint capsule, followed by the exposure of the hip joint for direct joint reduction (figure 1). We performed proximal femoral rotational osteotomy in cases with excessive anteversion or tension on the head, especially in older age groups. This osteotomy was carried out through the proximal femoral posterolateral approach. After appropriate femoral shortening and derotating, fixation of the osteotomy site was performed by a simple 4-hole plate. After that, iliac osteotomy by the Salter or Pemberton method (depend upon the femoral head size and acetabular capacity) through the previous hip approach was carried out. Open reduction of the femoral head in the acetabulum was achieved, then the joint capsule was reefed, and finally the wound closed in anatomical layers. Reduction was cheeked by flouroscopy and hip spica cast applied while the operated joint immobilized in the reduced position. The spica cast was removed after 1.5 months and radiographic control study was repeated every 6 months until the final visit.

## Results

Six children were excluded from the final study due to the previous surgical procedures. Among the remaining patients, six children (24%) were male and 18 children (76%) were female. Twelve children had bilateral dislocation and 12 unilateral dislocations (five with left hip and seven with right hip dislocation). Table 1 shows the frequency of Salter and Pemberton innominate osteotomy in our treated patients.

**Table 1:** The frequency of innominent osteotomy in treated patients

	**Salter innominate osteotomy**	**Pemberton acetabuloplasty**
Left side	15	1
Right side	14	6
Total	29	7

The mean age of the patients was 3.8±0.9 (range: 1.5-7). Most children (50%) were between two to four years old at the time of the surgery. Children with age <2 years and >6 years were 21% and 29%, respectively. We had five patients with associated clubfoot deformity and two with vertical talus. The mean follow-up period was 7.6±0.8 (range: 5.1-11.3) years. 

Preoperative radiographic reviews were assessed with *Tönnis* grade method that showed 14 hips as type IV, 15 as type III, and 7 as type II. The radiographs of operated hip joints were assessed by Severin classification system (table 2). The clinical results of children based on the age of presentation that have been evaluated by McKay’s classification are also depicted in table 3.

**Table 2:** Radiologic results according to Severin classification

**Severin classification**	**Class**	**No of hips**
Class I (Normal)	Ia	5
	Ib	7
Class II (Moderate deformity)	IIa	9
	IIb	11
Class III (Dysplasia without subluxation)	III	0
Class IV (Subluxation)	IVa	1
	IVb	2
Class V (Pseudoacetabulum)	V	0
Class VI (Re-dislocation)	VI	1

**Table 3:** Clinical results (McKay’s classification) based on the age of presentation

** **	<2 y/o*	2-4 y/o*	4-6 y/o*	>6 y/o*	**Total**
No of Children	5	12	5	2	24
Clinical Results					
-Excellent	3	2	1	0	6
-Good	1	7	3	1	12
-Acceptable	1	0	1	1	3
-Poor	0	3	0	0	3

*y/o: years old

**Figure 2:**
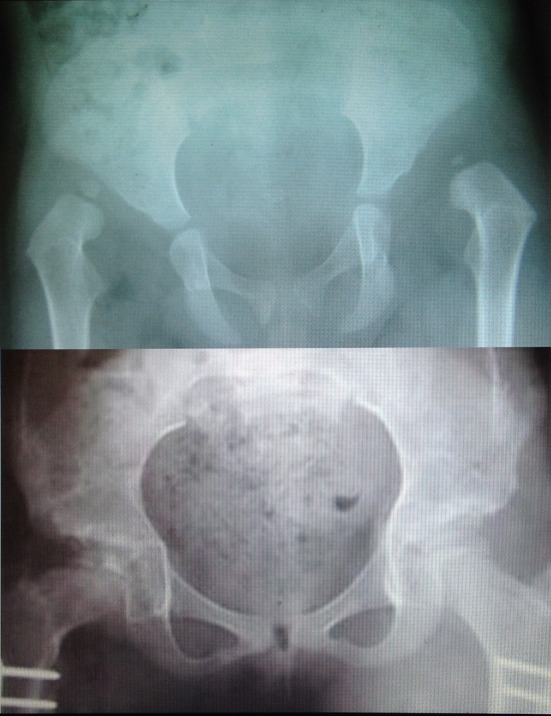
Above; a 2.5 year old girl with bilateral high hip dislocation. Below; eight years after bilateral Salter shortening, AVN and coxa vara developed on the right side.

Through the follow-up,two hips subluxated and AVN also occurred in three patients (figure 2). The age of these children at the time of the surgery was 2, 3.5, and 4 years old. We had also one re-dislocation three months after the primary operation. This child was operated again.

## Discussion

In this study, we evaluated the clinical and radiographic outcome of surgical treatment of DDH in walking age children. From a clinical point of view, 25% and 50% of our children could achieve excellent or good results, respectively and 61% of hips were radiographically good. According to the reports in orthopedic journals, although the success rate of open reduction with concomitant osteotomy of both the femoral and iliac bones reaches as high as 70% to 80%, this figure declines with longer-term follow-up.^20^ In this study, we had excellent and good results in 18 from 24 children (75%). Even though femoral shortening is reported to be necessary in all children more than three years of age, it might also be required in younger children when significant force is needed to reduce the hip joint.^21^ In this study, we performed femoral shortening in all cases irrespective of the age.

Combined osteotomy of the iliac and femoral bones for the treatment of DDH in children is a major and time-consuming surgical procedure that requires sufficient experience and precision. It is usually performed in ambulating children. To maintain joint reduced, variety of surgical methods have been invented. The indications for performing these operations depends on the type of dislocation and the size of the femoral head and acetabulum.^22^ There is no consensus regarding the application of a rotational osteotomy of the femur for correction of excessive neck anteversion. Some believe that, this type of correction is not necessary in a child who has recently started to walk and the stability test itself during the operation (as explained by Zadeh) can efficiently determine the necessity of the femoral rotational osteotomy.^23^ It seems that rotational osteotomy of the femur for correction of the neck anteversion is not required in unilateral dislocations with grade II and III of *Tönnis* classification. However, in Type IV group, this correction must be carried out to prevent re-dislocations after surgery.^24^

Magnetic resonance imaging of hips in children with unilateral DDH shows that the anteversion of the acetabulum is more than that of the femoral neck.^25^ The comparison between unilateral DDH with the normal contralateral side using computed tomography scan has also revealed that the degree of femoral neck anteversion of the affected side is more than the normal side.^26^ Some authors consider that the excessive pressure on the femoral head in the acetabular fossa is the main cause of re-dislocation after operation. Excessive femoral head pressures happen if anteversion of the femoral neck is not corrected and the pathologic tissue in the acetabular fossa is left behind. An increase in the acetabular index is also another factor leading to re-dislocation.^27^ Preoperative limb traction is no longer recommended due to the probable various complications such as AVN of the femoral head and redislocation.^28^ We did not use preoperative limb traction on any of our patients.

In a study by Rashid et al. at Robert Jones Hospital in England, the children who had undergone both open reduction and Salter osteotomy were assessed. The majority (83.8%) were categorized as grade I or II in the Severin classification. AVN and re-dislocation of hip joint requiring surgical intervention occurred in 8.1% and 5.4% of children, respectively.^29^ In this study, 88.9% of the conducted hip joints were graded as I or II, AVN occurred in three cases and re-dislocation in one.

In a recent study, Ting-Ming Wang et al. compared the outcomes of surgical treatment in unilateral and bilateral DDH in the children of walking age. The authors finally concluded that the clinical outcomes of bilateral DDH were worse than unilateral ones, primarily because of asymmetrical results. Age and Tönnis grade played an important role in the risk of AVN occurrence. The radiographic outcome according to the Severin classification did not differ significantly between the two groups.^30^ We did not compare clinical results of unilateral and bilateral dislocation in our patients in this study.

The limitations of the present study comprise its retrospective nature and the limited number of children. Yet, given the recent medical advances and the more possibility of earlier diagnosis and treatment, this number seems to be significant. 

## Conclusion

Although the concomitant osteotomies of both the iliac and femoral bones are time-consuming and may be associated with various complications, it is associated with satisfactory clinical and radiological outcomes. The success rate of these surgical procedures is considered acceptable and therefore recommended. 
